# Concomitant Posterolateral Elbow Dislocation with Ipsilateral Comminuted Intra-articular Distal Radius Fracture: A Rare Orthopaedic Scenario

**DOI:** 10.7759/cureus.2264

**Published:** 2018-03-03

**Authors:** Vivek Tiwari, Yugal Karkhur, Anupam Das

**Affiliations:** 1 Department of Orthopaedics, All India Institute of Medical Sciences, Bhopal, India; 2 Department of Orthopaedics, Maulana Azad Medical College, New Delhi, India; 3 Department of Orthopaedics, All India Institute of Medical Sciences, New Delhi, India

**Keywords:** distal radius fracture, elbow dislocation, trauma, colle's fracture

## Abstract

Fracture dislocations are common around the elbow joint. However, closed fracture of the distal radius with ipsilateral elbow dislocation is an uncommon injury pattern. We discuss the case of a middle-aged woman presenting with posterolateral elbow dislocation with concomitant ipsilateral closed intra-articular fracture of the distal radius. It was treated with closed reduction for the elbow dislocation first followed by closed reduction for the distal radius fracture. Even with conservative management, the patient had a good functional outcome at one year. The importance and incidence of such a rare injury pattern and the possible mechanism of injury has been discussed.

## Introduction

Distal radius fractures are one of the most commonly encountered trauma cases in any orthopaedic casualty [1]. The energy of the trauma causing such a fracture is dissipated either through distal radius fracture alone or in association with fracture dislocations in the nearby wrist joint. However, the association of distal radius fracture with concomitant ipsilateral postero-lateral elbow dislocation has been rarely described in the literature [2-4]. We present the case of a middle-aged female presenting with closed intra-articular distal radius fracture and ipsilateral postero-lateral dislocation of the elbow, which was managed with closed reduction of both the above injuries with good results.

## Case presentation

**Figure 1:**
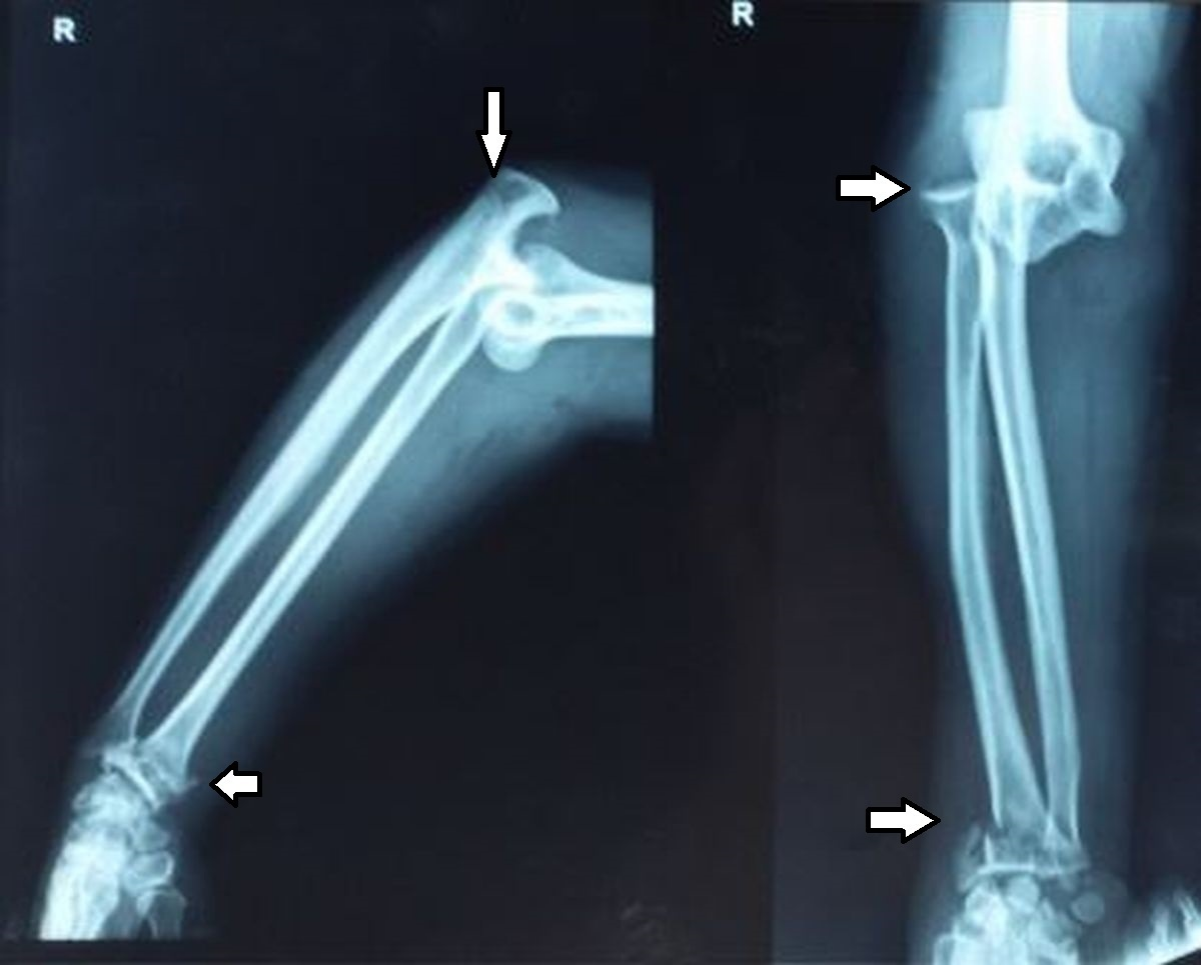
Pre-reduction plain radiograph of the right elbow and wrist, anteroposterior and lateral views Distal radius fracture with postero-lateral elbow dislocation (white arrows).

A 45-year-old housewife presented to the emergency department with complaints of pain and swelling in the right wrist and right elbow. She gave a history of fall from a bike on the outstretched hand in a high-velocity road traffic accident. There was no history of head injury or injury to any other part of the body. On examination, there was swelling and deformity in the right wrist as well as the right elbow. The right wrist joint was tender and the range of movement was painful. Similarly, there was tenderness in the right elbow and range of movement could not be assessed due to the pain. Crepitus could be felt at the right wrist joint suggesting a fracture, whereas there was no crepitation in the elbow. The distal neurovascular status was normal. Plain radiographs revealed severely-comminuted distal radius fracture with dorsal angulation with intra-articular extension, along with concomitant ipsilateral postero-lateral elbow dislocation (Figure 1).

**Figure 2:**
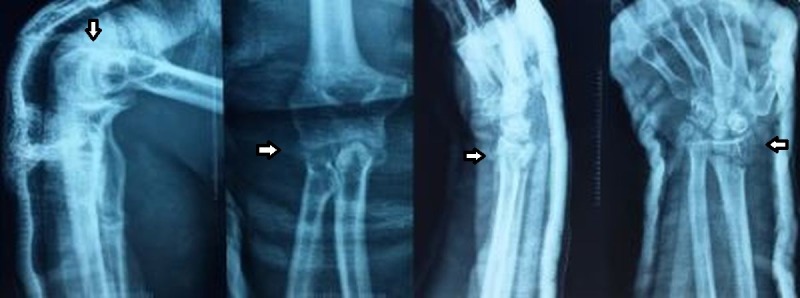
Post-reduction plain radiographs of the right elbow and wrist, anteroposterior and lateral views Congruent reduction of the elbow joint and acceptable reduction of the severely comminuted distal radius fracture with intra-articular extension (white arrows).

Closed reduction of the right elbow was done first in the emergency room under sedation with longitudinal traction and flexion of the elbow. The distal radius fracture was then reduced by giving longitudinal traction followed by initial dorsal tilt to dis-impact the fracture and then volar and ulnar tilt. The distal neurovascular status was again checked immediately after closed reduction and was found to be intact. The check films showed a congruent reduction of the elbow joint along with an acceptable reduction of the distal radius fracture (Figure 2).

However, the patient was counselled for pinning of distal radius fracture in view of severe comminution and intra-articular extension to prevent a longitudinal collapse of the distal fragment. Due to financial constraints, the patient refused any operative intervention and hence was managed with an above-elbow posterior slab for one week followed by an above-elbow cast for three weeks. She was given a below-elbow cast for further two weeks. Active elbow and wrist range-of-motion exercises were started after six weeks of trauma. At the follow-up of one year, she had a good functional outcome with painless, full extension and flexion movements of the wrist and the elbow joint as well as full range of pronation and supination. She could resume her household work without any functional deficit.

## Discussion

Fracture dislocations are common around the elbow joint in skeletally mature population. However, the usual fractures involved are those of proximal ulna or radius [5]. Elbow dislocation with concomitant diaphyseal fractures of both radius and ulna are also common [6]. On the other hand, fracture of the distal radius with ipsilateral elbow dislocation is an uncommon injury pattern. Only a few cases were found with such combination on an extensive search of the literature. Most of the reported cases were either described in children or had compounding of the distal radius fracture or elbow [2-3]. The only other case of a closed distal radius fracture with ipsilateral posterior elbow dislocation that has been reported in an adult did not have comminution in the distal radius [4]. This is the first report of a conservatively managed, severely comminuted, intra-articular distal radius fracture with concomitant ipsilateral postero-lateral elbow dislocation occurring in a high-velocity road traffic accident.

The mechanism of injury probably involved longitudinal compressive force with an extension (dorsal) component as the fracture had dorsal angulation. Therefore, the reduction should logically be attempted in flexion of the wrist as well as the elbow joint with longitudinal traction. In the previously reported case, the elbow dislocation was reduced in extension whereas, in our case, it was reduced in flexion [4]. The reduction of both components in the same direction increases the ease of such manipulation in the busy emergency room. In our case, proper initial reduction of both the components resulted in a good functional outcome even with conservative management, thereby underlining the importance of good reduction manoeuvre in the emergency room.

## Conclusions

The above case underscores the importance of thorough assessment of the wrist using clinical examination and if required, plain radiographs in cases of elbow dislocation, particularly in high energy trauma. This case report will increase awareness about the possibility of such a combination and will help in preventing missed injuries.

## References

[1] Meena S, Sharma P, Sambharia AK, Dawar A (2014). Fractures of distal radius: an overview. J Family Med Prim Care.

[2] Batra S, Andrew JG (2007). Ipsilateral compound distal radius fracture with missed elbow dislocation. A rare injury pattern. Eur J Emerg Med.

[3] Nanno M, Sawaizumi T, Ito H (2007). Transverse divergent dislocation of the elbow with ipsilateral distal radius fracture in a child. J Orthop Trauma.

[4] Meena S, Trikha V, Kumar R, Saini P, Sambharia AK (2013). Elbow dislocation with ipsilateral distal radius fracture. J Nat Sci Biol Med.

[5] Ring D, Jupiter JB (1998). Current concepts review: fracture-dislocation of the elbow. J Bone Joint Surg Am.

[6] Hung SC, Huang CK, Chiang CC, Chen TH, Chen WM, Lo WH (2003). Monteggia type 1 equivalent lesion: Diaphyseal ulna and radius fractures with a posterior elbow dislocation in an adult. Arch Orthop Trauma Surg.

